# International Comparison of Qualification Process for Medical Product Development Tools

**DOI:** 10.1007/s43441-024-00630-9

**Published:** 2024-03-27

**Authors:** Daichi Uchijima, Shingo Kano

**Affiliations:** Bio-Innovation Policy Unit, Department of Computational Biology and Medical Sciences, Graduate School of Frontier Sciences, University of Tokyo, Bioscience Bldg B1-17, 5-1-5, Kashiwanoha, Kashiwa City, Chiba 8562 Japan

**Keywords:** Development tool, Qualification, Guidance of guidances, Process definition, International comparison

## Abstract

**Introduction:**

Qualification of medical product evaluation tools is underway in the United States, Europe, and Japan to reflect the advancements in the basic science of medical products. In Europe and the U.S., Guidance of Guidances (GoG) policies that clarify regulators’processes, tasks, and methods of sponsor involvement are adopted to issue tool guidance. However, in Japan, a non-GoG type policy focusing on supporting the research and development for tools without defining a tool guidance-making process has been adopted.

**Methods:**

In this study, an analytical framework for the lifecycle of development tools was constructed, including pre- and post-tool qualification processes, to compare the two above-mentioned approaches. For this study, Japanese cases were selected as experimental cases, whereas Western cases served as controls. The progress of tool qualification and composition of deliverables were analyzed.

**Results and Conclusions:**

It was indicated that in the GoG type policy, in which processes are defined, and involvement methods are clarified, tool qualification can progress more smoothly than in a non-GoG type policy. This policy indicates that deliverables may have a consistent composition. Contrastingly, GoG-type policies alone present challenges in connecting upstream tools for R&D support.

## Introduction

### Importance of Development Tool Qualification

The Critical Path Initiative (CPI) [1]was launched in the U.S. in March 2004 as a national strategy by the Food and Drug Administration (FDA) to transform the development, evaluation, and manufacturing processes of regulated medical products. As part of this initiative, a document titled “Innovation/Stagnation: Challenge and Opportunity on the Critical Path to New Medical Products” was released [2], which advocates modernization of evaluation methods collectively referred to as “development tools,” emphasizing the need for collaborative efforts to evaluate and predict the safety, efficacy, and manufacturability of medical products.

The FDA and European Medicines Agency (EMA) have initiated Voluntary Pharmacogenomic Data Submissions (VGDS) and also joint FDA-EMA VGDS briefing meetings, in which sponsors voluntarily submit pharmacogenomic data to begin the process [3]. Through these initiatives, they have established a “Tool Qualification System” to assess Drug Development Tools (DDTs) [4]. Similar to the Drug Development Tool Qualification Program (DDTQP) launched in the U.S. [5], the Qualification of novel methodologies for medicine development (QNM) was initiated in Europe [6].

Led by the FDA and EMA, the Japan Pharmaceuticals and Medical Devices Agency (PMDA) started Consultations on Pharmacogenomics/Biomarkers (CPB) [7]. However, CPB should be interpreted differently from Western tool qualification systems, such as the DDTQP and QNM, as its main purpose is individual consultation on submission, and the disclosure of qualification results is not mandatory.

Based on the efforts toward DDT, a similar system has been established for medical devices. In the U.S., the FDA clarified the qualification process of the Medical Devices Development Tool (MDDT) and established the Medical Device Development Tool Qualification Program (MDDTQP) [8].

The FDA states that through the public release of development tool guidance based on qualifications, sponsors will be able to utilize the tool, thus helping to optimize evaluation in medical product development by increasing availability of effective drugs, enabling early access to medical treatment, and enhancing knowledge about investigational drugs [9]. Numerous applications for tool qualifications have been submitted, and the ongoing FDA DDT project currently encompasses 163 projects. The industry also highlights the lack of tools, such as patient selection tools during clinical trials, as a significant challenge in drug development, emphasizing the potential of tool qualifications to facilitate the advancement of the medical product development process [10–12].

Previous studies have reported tool qualifications using the DDTQP. Miller et al. of the Chronic Obstructive Pulmonary Disease (COPD) Biomarker Qualification Consortium (CBQC) summarized and reported their efforts to qualify plasma fibrinogen as a prognostic biomarker for patient selection in COPD clinical trials using the DDTQP [10]. This case report presents a timeline of the DDT qualifications, communication with regulatory authorities, and the content of the submitted data. It describes the activities within the DDTQP and explains the benefits of tool qualifications and usefulness of the DDTQP.

### International Comparison of Tool Qualification Systems

Walker et al. compared the policies and activities of the “Tool Qualification System” in the FDA, EMA, and PMDA from three perspectives to demonstrate that voluntary submission contributes to the governance of tool utilization [4]. First, they compared the policies in the qualification phase, review team, public consultation period, scope, fees, and regulatory products. Second, they compared the total number of tool qualification applications and qualifications by stage in each country. Third, specific examples of descriptions of Context of Use (COU) were analyzed as case studies. The study reported that, while there were differences in policies across countries, voluntary submission has driven tool qualification systems from cases where qualified tools have led to faster decision-making in diagnostics and have been useful for subject selection in clinical trials. Regarding the intended contribution of qualified tools aimed at evaluating medical products in each country’s clinical studies, it was concluded that although there are currently only few qualified tools, further consideration is needed and the policy will evolve as the field matures.

They claimed that voluntary submission facilitated tool qualification. However, the authors did not evaluate the impact of voluntary submission on tool qualification by comparing the presence or absence of voluntary submission. Although they set multiple criteria for institutional comparison, they did not thoroughly examine the process of tool qualification, thereby neglecting to analyze the influence of grant availability before process initiation and the specific content of qualification. Furthermore, the Japanese CPB case differs from the “Tool Qualification System” in Europe and the U.S. as it does not disclose tool guidance. Thus, it did not fall under the category of tool qualification, raising doubts regarding the validity of the case selection.

Similarly, a research report by Kshatriya et al., affiliated with Gujarat Technological University, conducted a comparative institutional analysis of tool qualification systems, including a Japanese case. They compared the qualification processes for DDT in the U.S., Europe, Japan, and India [13]. They introduced the policy content of each country and compared the policies on five aspects: procedure, scope, applicant, when to submit, and how to submit them. They concluded that while Europe and the U.S. have established a clear understanding of the policy framework for DDT, India’s framework is inadequate. In Japan, they noted a lack of information and did not provide specific comments. The cases selected in Japan included CPB, companion diagnostics, and ethical regulations for animal testing, raising concerns about the appropriateness of the case selection. Additionally, there are several factual inaccuracies, such as the assertion that the Japanese application method for tool qualification is the same as that in the U.S. and that the process is free of charge.

A common issue observed in these international comparisons is that the selected case from Japan does not correspond to the tool qualifications, leading to an inaccurate analysis.

### Tool Qualification System as a Guidance of Guidances

Tool qualification programs, such as the DDTQP, MDDTQP, and QNM, qualify as voluntary submitted development tools and issue tool guidance based on clearly defined processes and procedures. Therefore, these systems can be characterized as guidance for issuing tools. The U.S. FDA staff are required to adhere to Good Guidance Practices (GGP), referred to as the Guidance of Guidances (GoG) [14], which provides a guidance-making process and compliance requirements for U.S. FDA staff. The 2011 GGP report (2011) defines the stages of the guidance lifecycle as follows: (1) Initiating Guidance (i.e., the decision to begin developing guidance), (2) Prioritizing/Work Planning/Tracking Guidance, (3) Developing Guidance, (4) Reviewing and Clearing Guidance; and (5) Issuing Guidance and Outreach [15]. While DDTQP/MDDTQP divides the process into three phases: (1) initiation, (2) consultation, and (3) review, the contents of the five stages of the GGP are included and correspond to each other. Although the GGP serves as a higher-level concept, the difference between the GGP and DDTQP lies in the more detailed definition of the pre-evidence review process in DDTQP [16].

The tool qualification system in Japan is characterized by not adopting a GoG type tool guidance creation system as in Europe and the U.S. and lacks a well-defined process. However, existing international comparative studies have not considered these differences in analytical methods. Therefore, to compare tool qualification systems internationally, including Japan, we developed an analysis method that defines the process in a GoG type general framework, allowing for the analysis of both GoG and non-GoG type qualification systems. Additionally, we included observations and analyses of the processes before and after the guidance lifecycle to capture a broader perspective.

## Research setting and Framework

### Analytical Framework for Tool Qualifications

To analyze the tool qualification process, we constructed an analysis framework by breaking down the tool lifecycle, including the timelines before and after tool qualification process (Table 1). Furthermore, we included the agencies responsible for each step of the tool-qualification process (Table1).

**Table 1:**
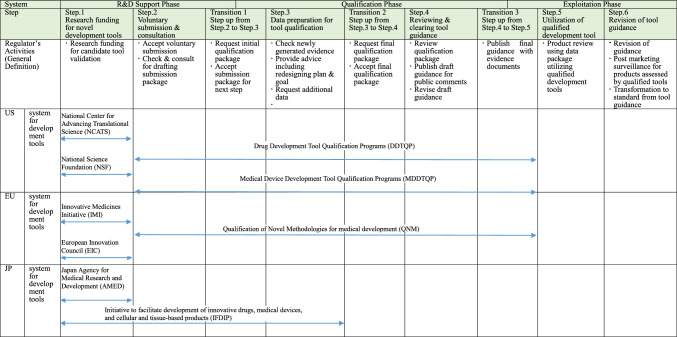
Analytical Framework for Regulator’s Activities based on the tool lifecycle

For Step.1 support only, list the name of the organization: NCATS, NSF, IMI, EIC, AMED

The broader tool qualification process comprises three main phases: (R&D) Support Phase, which supports tool development through grants and other means; Qualification Phase, which involves development of tool guidance; and Exploitation Phase, which involves the actual use of the tool. Each phase was further divided into Steps 1–6.

In the R&D Support Phase, Step 1 is “Research Funding Support for New Tools” and Step 2 is “Voluntary Submission and Consultation.” In the Organization for Economic Co-operation and Development (OECD)’s TEST GUIDANCE flow, the first two steps correspond to “TEST development” and “initial steps of the validation process,” indicating the transition from the research and development stage to pre-validation [17]. Pre-validation includes activities such as optimization and reproducibility, which are part of the qualification process. However, there may be some overlap between the activities during the research and development stages. Step 1 represents the R&D stage and Step 2 represents the stage in which the qualification process is initiated through voluntary submission.

In the Qualification Phase, Step 3 is “Data Preparation for Tool Qualification” and Step 4 is “Reviewing & Clearing Tool Guidance.” In DDTQP [16], MDDTQP [18], and QNM [19], before entering the review process, there is a stage in which the innovator and regulator confirm the data necessary for the application package. This stage is described in Step 3. Once the regulator accepts the application package, the process of review, public comment solicitation, and revisions occur, which are represented in Step 4.

In the Exploitation Phase, Step 5 is “Utilization of Qualified Development Tool” and Step 6 is “Revision of Tool Guidance.” In clinical trials, the tool is used for product evaluation and approved products are reviewed through post-marketing surveillance after approval. During this process, the development tool was utilized based on tool guidance (Step 5). As official standards, such as those of the International Organization for Standardization (ISO), are regularly reviewed [20], tool guidance is also subject to review, which may include the evolution of tool guidance toward ISO standards (Step 6).

“Transition” refers to the transition between steps that involve regulator’s judgments. Transition 1 represents the transition from Steps 2 to 3, involving a regulatory acceptance decision regarding the initial tool guidance package submitted by the tool inventory/provider. Transition 2 represents the transition from Steps 3 to 4, involving a regulatory acceptance decision regarding the final tool guidance package submitted by the tool inventor/provider. Transition 3 represents the transition from Steps 4 to 5, involving the final regulatory acceptance or rejection decision after considering the public comments on the tool guidance draft. Defining the processes in “Transition” clearly is a significant feature of GoG type systems. The DDTQP [16], MDDTQP [18], and QNM [19] explicitly describe the processes related to these “Transitions” in their guidance documents. The 2014 version of the DDTQP guidance includes a flowchart illustrating these transitions, which was updated in 2020 [21].

### Method for Comparing the Composition of Deliverables in Tool Qualification Activities

To compare the composition and content of deliverables in tool qualification activities, an analysis was conducted on the types of deliverables, types of tools addressed, contexts of use (COU) for the tools, and the content of evidence provided in the COU for the tools.

### Case Selection

#### Japanese Cases

When selecting cases for tool qualification in Japan, it is necessary to select a system that subsidizes the research and development of evaluation methods and covers activities up to the release of the tool guidance. Therefore, it is necessary to select a grant program that meets the following three conditions:

##### Separation of Product and Tool

In many cases, the program supports R&D for the entire target product; therefore, it is essential that only the R&D activities for tool evaluation be observed separately.

##### Guidance Formulation Process

The project objectives must include not only the research and development of the tool but also the issuance of tool guidance, and the activities up to the issuance of tool guidance must be observable. However, the case selection should include cases that have not yet reached the tool guidance issuance stage.

##### Information Disclosure

A detailed disclosure of project data should be available, which is consistent with the analytical framework of this study.

In Japan, the only grant program that meets these conditions is the “Initiative for Facilitating the Development of Innovative Drugs, Medical Devices and Cell/Tissue-derived Products (IFDIP) [22, 23],” and among the IFDIP projects (10 in the pharmaceutical field, seven in the medical device field, and seven in the regenerative medicine and other products field), four projects were selected because products and tools can be separated and categorized as follows (Table 2):

1. Case that issued tool guidance during the IFDIP Project term (one case)
2. Case that issued tool guidance after the IFDIP Project term (one case)
3. Cases that published opinion papers instead of tool guidance during the IFDIP Project term (two cases)

**Table 2:** Profile of selected cases

Case Category	Case Tool	Project Description	Team Composition*
Tool Guidance issued within the IFDIP Project term	Coronary stent durability test method	Research to establish a non-clinical performance evaluation system and evaluation method based on Engineering Based Medicine	Tokyo Women’s Medical Univ. -Waseda Univ. Joint Institution for Advanced Biomedical Sciences (TWIns), NIHS, PMDA, other univ
Tool Guidance issued after the IFDIP Project term	Platelet product quality evaluation	Research to establish evaluation methods for iPS cells for regenerative medicine	Center for iPS Cell Research and Application, Kyoto Univ. (CiRA), NIHS, PMDA, other univ
Opinion Paper published instead of tool guidance	Pharmacokinetic evaluation using genetic polymorphism of drug metabolizing enzymes and transporters	Genomic pharmacology, research on evaluation methods for efficacy and safety of drugs using biomarkers	Tohoku Univ., NIHS, PMDA, CRO, other univ
	Biomarkers used for clinical trial patient selection criteria and efficacy evaluation for Alzheimer’s Disease (AD)	Regulatory science research for the establishment of criteria for clinical evaluation of drugs for Alzheimer’s Disease	Univ. of Tokyo, PMDA, hospital, other univ
Control Case: Tool Guidance issued by DDTQP	Plasma fibrinogen for predicting exacerbation of chronic obstructive pulmonary disease (COPD)	The biomarker Plasma Fibrinogen, in interventional clinical trials of patients with chronic obstructive pulmonary disease (COPD) at high risk for exacerbations and/or all-cause mortality	Chronic Obstructive Pulmonary Disease (COPD) Biomarker Qualification Consortium (CBQC)

*University (Univ.), National Institute of Health Sciences (NIHS), Pharmaceuticals and Medical Devices Agency (PMDA)

Data were sourced from the IFDIP website and e-Gov Japan [24].

#### U.S. Cases as Controls

As a control case representing a GoG type system for comparison with the non-GoG Japanese case, we selected a common European and U.S. case qualified by the DDTQP in the United States. Other selection criteria were that R&D activities in the upstream part of the eligibility process should be identifiable and that a detailed report by the applicant should exist. The selected case involved a Plasma Fibrinogen biomarker submitted by the Chronic Obstructive Pulmonary Disease (COPD) Biomarker Qualification Consortium (CBQC) [9, 10].

## Results

### Process Based Analysis

Table 3 presents the results of the step-by-step analysis of each case based on the framework.

**Table 3:** Comparison of IFDIP and DDTQP cases

System	R&D Support Phase			Qualification Phase				Exploitation Phase		
Step	Step.1 Research funding for novel development tools	Step.2 Voluntary submission & consultation	Transition 1 Step up from Step.2 to Step.3	Step.3 Data preparation for tool qualification	Transition 2 Step up from Step.3 to Step.4	Step.4 Reviewing & Clearing tool guidance	Transition 3 Step up from Step.4 to Step.5		Step.5 Utilization of qualified development tools	Step.6 Revision of Tool guidance
Case										
Regulator’s Activities (general definition)	-Research funding for candidate development tool validation	-Accept voluntary submission -Check & consult for drafting submission package	-Request initial qualification package -Accept submission package for next step	-Check newly generated evidence -Provide advice including redesigning plan & goal -Request additional data	-Request final qualification package -Accept a final qualification package	-Review qualification package -Publish draft guidance for public comments -Revise draft guidance	-Publish final guidance with evidence documents		-Product review using data package utilizing qualified development tools	Revision of guidance -Post marketing safety measures -Transformation to standard from tool guidance
Plasma fibrinogen / CBQC	-Sponsored by the COPD Foundation, NIH/ National Heart, Lung and Blood Institute [25]	-Received a letter of intent (LOI) to the FDA describing DDT profile, proposed COU and importance of Plasma fibrinogen for COPD and available data by the submitter in 2011 [10] -Checked and consulted for LOI and available data [10]	-Accepted the LOI in 2012 -Created Biomarker Qualification Review Team (BQRT). [10]	-Determined the threshold of plasma fibrinogen level as a predictive biomarker for COPD exacerbation [10] -Received a final qualification package in 2013 [26]	-Accepted final qualification package in 2014 [10]	-The FDA reviewed the qualification package in 2015 -FDA issued its draft guidance in 2015 -No Public comment [27]	-Published final guidance with evidence document in 2016 [28]		-Product reviewed using data package utilizing qualified development tools in 2020 [29]	-Expanded COU as a diagnostic biomarker in 2021 [30]
Coronary stent durability test method / TWIns	-Received an IFDIP proposal in 2012 [32] -Fund by IFDIP of MHLW/PMDA in 2012 [32] -Built PJ team including PMDA staff in 2012 [32]	-Checked and advised a proposal of guidance through PJ team meeting and annual reporting of IFDIP in 2013 [32]	-Made a consensus within a PJ team including PMDA for a proposal of guidance in 2013 [33]	-Made a consensus within a PJ team including PMDA for additional data acquisition in 2014 [33] -Checked comments from outside specialists, industries, NIHS in 2014 [33] -Received the final proposal from the PJ team in 2014 [34]	-Accepted a final proposal in 2014 [35]	-Reviewed in MHLW [36] -MHLW issuing its draft guidance for public comments in 2015 [36] -9 public comments in 2015 [36] -Revised draft guidance in 2016 [36]	-Published final guidance in 2016 [33]		-Not disclosed	-(Transformed JIS T0402 by innovator) [37]
Platelet product quality evaluation/CiRA	-Received an IFDIP proposal in 2012 [38] -Funded by IFDIP of MHLW/PMDA in 2012 [38] -Built PJ team including PMDA staff in 2012 [38]	-Checked and advised a proposal of guidance through PJ team meeting and annual reporting of IFDIP in 2014 [39]	-Made a consensus within a PJ team including PMDA for a proposal of guidance in 2014 [39]	-Made a consensus within a PJ team including PMDA for additional data acquisition in 2014, 2015 [40] -Checked comments from outside specialists, association, and industries in 2016 [41]	-Not disclosed	-(Shadow process) Reviewed in MHLW [42] -MHLW issuing its draft guidance for public comments in 2017 [42] -9 public comments in 2018 [42] -Revised draft guidance [42]	-Published final guidance in 2018 [43, 44]		-Not disclosed	-N/A
Pharmacokinetic evaluation using genetic polymorphism of drug metabolizing enzymes and transporters/Tohoku University	-Received an IFDIP proposal in 2012 [45] -Funded by IFDIP of MHLW/PMDA in 2012 [45] -Built PJ team including PMDA staff in 2012 [45]	-Checked and advised a proposal of guidance through PJ team meeting and annual reporting of IFDIP in 2013 [46]	-Made a consensus within a PJ team including PMDA for a proposal of guidance in 2014 [46]	-Made a consensus within a PJ team including PMDA for additional data acquisition in 2014, 2015 [46] -Checked comments from outside specialists, association, and industries in 2016 [46] -Published opinion paper in 2016 [47]	-Not disclosed	-N/A	-N/A		-N/A	-N/A
Biomarkers used for clinical trial patient selection criteria and efficacy evaluation for Alzheimer’s Disease (AD) / The University of Tokyo	-Received an IFDIP proposal in 2012 [50] -Funded by IFDIP of MHLW/PMDA in 2012 [50] -Built PJ team including PMDA staff in 2012 [50]	-Checked and advised a proposal of guidance through PJ team meeting and annual reporting of IFDIP in 2013 [48]	-Made a consensus within a PJ team including PMDA for a proposal of guidance in 2013 [48] -Released interim deliverable in 2013 [48]	-Make a consensus within a PJ team including PMDA for additional data acquisition in 2014, 2015 [48] -Checked comments from FDA, EMA, outside specialists, association, and industries in 2015, 2016 [48] -Published opinion paper in 2016 [51]	-Not disclosed	-N/A	-N/A		-N/A	-N/A

#### Control Case in US: Plasma Fibrinogen for COPD

This Project (hereafter PJ) received funding from the COPD Foundation and the NIH/National Heart, Lung and Blood Institute [25]. In Step 2, the CBQC submitted a Letter of Intent (LOI) to the FDA, which was accepted by the FDA, leading to Transition 1 [10]. At this stage, the FDA formed the Biomarker Qualification Review Team (BQRT). In Step 3, the CBQC submitted the final qualification package to the FDA based on consultations with the BQRT [26]. The FDA determined that the final qualification package had no issues (Transition 2). Subsequently, in Step 4, a draft guidance was developed by the FDA and entered the public comment phase [10]. As no comments were received on the draft guidance, it was finalized and issued as official guidance, leading to Transition 3 [27, 28]. Plasma fibrinogen was used in the clinical trial inclusion criteria in Step 5, indicating its utility for regulatory submissions [29]. Furthermore, research is being conducted to expand the clinical COU of plasma fibrinogen from a Prognostic Biomarker to a Diagnostic Biomarker, leading to ongoing revision activities for tool guidance, progressing to Step 6 [30].

#### A Case of Tool Guidance Issued Within a Project Term: Quality Evaluation Tool for Coronary Stents

The regulators’ Step 1 activities are common in each IFDIP case. After the PMDA receives a submission to the IFDIP, if the submission is adopted, it organizes a project team incorporating the PMDA staff and implements funding for four years [31].

Starting in early 2012, the Tokyo Women’s Medical University-Waseda University Joint Institution for Advanced Biomedical Sciences (TWIns) was proactive in its guidance development activities with a concrete plan for drafting tool guidance. Later in the same year, a working group for tool guidance development was initiated [32]. In 2013, as part of Step 2 activities, an outline of tool guidance was created after consulting regulators within the PJ team and proceeding to Transition 1 [33]. In 2014, the PJ team formed a consensus on the data necessary for tool guidance and created a final package incorporating opinions obtained from external experts, industry organizations, and the National Institute of Health Sciences (NIHS) (Step 3). The final package was submitted to the MHLW [34]. The MHLW confirmed the adequacy of the final package and accepted it, thus completing the submission and acceptance processes (Transition 2) [33, 35]. In 2015, in Step 4, the MHLW reviewed the accepted final package and issued draft guidance [36], that received nine comments during the public comment period. Based on these comments, the draft guidance was revised and officially issued as tool guidance in 2016 [33]. Because the tool is used in non-clinical trials, it is difficult to confirm its use in clinical trial reports, and no information about Step 5 activities could be found. However, as part of Step 6 activities for this tool, although initiated by the technology holder, a transformation to Japanese Industrial Standards (JIS) was conducted [37].

#### A Case of Tool Guidance Issued after Project Termination: Quality Evaluation for Platelet Product

The Center for iPS Cell Research and Application at Kyoto University (CiRA) initiated Step 1 in 2012 [38]. However, document submission reviews and consultations for Step 2 began in 2014 [38, 39]. According to the PJ report, as of 2013, the outline of the tool guidance was completed by the CiRA, the representative of the PJ team. However, discussion within the PJ team was delayed, resulting in a consensus among the PJ team, including the PMDA, on Transition 1, which was reached in 2014 [39]. Step 3 activities, including the confirmation of additional evidence data and consensus-building within the PJ team, including the PMDA staff, took place until 2015. In 2016, external experts, industry organizations, and relevant academic societies were consulted and the PJ term dissolved [39–41]. The activities after the IFDIP PJ term were not publicly disclosed; however, in 2017, a draft guidance was issued by the MHLW [42]. The draft guidance underwent a public comment period and received nine comments. Based on these comments, the draft guidance was revised and officially issued as tool guidance in 2018 [43, 44].

#### A Case of Opinion Paper Issued Other than Tool Guidance: Pharmacokinetic Evaluation Using Genetic Polymorphism of Metabolizing Enzymes and Transporters

Tohoku University initially considered the preparation of draft guidance as part of the goal of the IFDIP PJ [45, 46]. However, in Step 3, an opinion paper was published as the basis for developing the draft tool guidance, reflecting input from external experts, relevant academic societies, and industry organizations, along with the final package created by the PJ team [47]. This review outlines the evaluation methods for the impact of genes and their polymorphisms on the metabolism and transport activity of investigational drugs, including analytical methods for multiple target genes. Activities after the PJ term were not confirmed, and there was no record of any submissions made to the MHLW regarding the issuance of tool guidance.

#### A Case of Opinion Paper Issued Other than Tool Guidance: Biomarkers Used for Clinical Trial Patient Selection Criteria and Efficacy Evaluation for Alzheimer’s Disease (AD)

The University of Tokyo considered the preparation of draft guidance to be a goal of the IFDIP PJ and planned to issue tool guidance within one year after the conclusion of the IFDIP [48, 49]. Furthermore, in Transition 1, the PJ team submitted an interim report to the MHLW/PMDA, which was made publicly available. In Step 3, the PJ team revised the publicly available interim report and created an opinion paper, which was then released by the MHLW following input from the FDA, EMA, external experts, relevant academic societies, and industry organizations, [50, 51]. This opinion paper outlines patient selection methods during clinical trials for early intervention in Alzheimer’s and the utilization of surrogate endpoints such as the COU, including multiple clinical evaluation methods involving biomarker assessments. There is no confirmed record of any submissions to the MHLW regarding the issuance of guidance tools after the PJ term.

### Analysis of Components of Guidance or Final Report

Table 4 presents the results of the analysis of the guidance content in terms of deliverable type, target tool type, COU of the tool, and content of evidence in the COU of the tool.

**Table 4:** Components Analysis of DDTQP and IFDIP deliverables

Principle of PJ team	Tool (abbreviation)	Abbreviated Context of Use	Composition of tool guidance/final package
CBQC [26, 28]	Plasma Fibrinogen	Prognostic biomarker used with other characteristics to enrich for COPD exacerbations as patient inclusion criteria in interventional clinical trials	Type: Single Tool guidance 1. Tool specification 2. COU: Use Statement for single disease 3. Conditions for qualified use with evidence data
TWIns [37]	Coronary stent durability test method	Non-clinical test method to evaluate the long-term durability of permanently implanted coronary stents using simulated blood vessels	Type: Single tool guidance 1. Tool specification 2. COU: Scope of application for quality of single product 3. Details of test methods with reference
CiRA [44]	Platelet product quality evaluation	Quality testing methods for the manufacture of human iPS cell-derived differentiation-inducing platelet products (Impurity test, functional evaluation test, endotoxin test, evaluation of residual amounts of additives/impurities, mycoplasma negativity test, sterility test, virus negativity test, etc.)	Type: Multiple tools guidance 1. Tool specification 2. COU: Scope of application for quality of single product 3. Evaluation items, notes/recommendations with Reference
Tohoku University [47]	Pharmacokinetic evaluation using genetic polymorphism of drug metabolizing enzymes and transporters	Methods for evaluating the effects of genes and their polymorphisms on the metabolic and transport activities of test drugs	Type: Scientific recommendation for single method 1. Tool specification 2. COU: Scope of application for multiple genes 3. Enumeration of analysis methods with Reference
The University of Tokyo [51]	Biomarkers used for clinical trial patient selection criteria and efficacy evaluation for Alzheimer’s Disease (AD)	Patient selection methods and use as surrogate endpoints during clinical trials for Alzheimer’s disease early treatment intervention	Type: Scientific recommendation for multiple tools 1. Tool specification 2. COU: Scope of application for single disease 3. Enumeration of analysis methods with Reference

#### Control Cases

The guidance for plasma fibrinogen (proposed by CBQC) is a common DDTQP single tool type document for single disease and published as a document titled “Biomarkers Qualification Review for Plasma Fibrinogen” [26, 28]. It describes the background, tool profile, COU, data sources, major findings, data considerations, BQRT conclusions, and BQRT recommendations for tool qualification and serves as an evidence document for tool qualification.

#### IFDIP Cases

The guidance for the coronary stent durability test (proposed by TWins) is only a single tool-type document for a single disease in the IFDIP, although clarifying the tool and describing the COU in the guidance does not directly include evidence data and only adds reference standards and references [37].

The Guidance for Platelet Product Quality Evaluation (proposed by the CiRA) is a multiple-tool-type document for the quality of a single product that organizes recommendations for platelet product quality assurance. The guidance describes the scope of application, evaluation items, and precautions/recommendations for each test as a COU to evaluate the quality of platelet products. However, it does not mention specific test conditions, number of specimens, procedures, etc., for each test and only provides reference information. In this guidance, there is no publication of evidence documents, reference standards, or literature [44].

Tohoku University and the University of Tokyo have issued opinion papers that outline scientific information and considerations for product evaluation, although they differ in terms of the combination of the COU and the tool. Tohoku University’s opinion paper focused on the use of a single method for multiple genes, whereas The University of Tokyo’s opinion paper discussed the use of multiple tools for one disease. The specific testing methods were left to the discretion of the testers. Neither document provided a direct presentation of evidence data but instead included reference standards and literature [47, 51].

## Discussion

### Validation of the Analytical Framework

Using the analytical framework developed in this study, we observed the progress of each step in the process, along with its activities, by analyzing the CBQC plasma fibrinogen as a control. The control case covered the entire process defined in the framework, demonstrating its applicability to the lifecycle analysis of development tools. Consequently, using this framework, it was possible to profile the regulator’s activities in each process of the comparative control case qualified by the GoG system and confirm that it could serve as a reference for the description of each process when analyzing qualification cases in non-GoG systems.

### Rationale for Process Definition

The case of TWIns revealed that even in the absence of a process definition, they proceeded with Transition 2 and subsequent steps that were not covered by the IFDIP. It was inferred that they voluntarily conducted the activities required for each process after Transition 2 within the PJ team, allowing them to create guidance during the PJ term. To perform the activities required for tool qualification without a process definition or a review team to serve as a regulator’s point of contact, it is essential that the applicant has knowledge and experience of what is required in each process and the procedures to proceed to the next process. It is also necessary to have a human network to check the details of the implementation with a regulator. In fact, Waseda University, to which TWIns is partially affiliated, has the Institute for Medical Regulatory Science (IMeRS) and has extensive regulatory expert networks and experience in regulatory efforts. For this reason, it is interpreted that TWIns members were able to leverage the knowledge accessible within the network to conduct the activities required to qualify the tools [52].

The CiRA case provides tool guidance. However, it took two years to transition from Step 1 to Step 2, causing a delay in initiating subsequent steps, and the activities after Transition 2 remained incomplete within the IFDIP Project term. If explicit procedures for Steps 1 and 2 had been defined, the stagnation of activities could have been avoided.

The two university cases revealed that the activities in each process up to Step 3 were almost identical to those in the control case. These two cases aimed to make the guidance draft guidelines within the study period. However, there were no records of progression to Transition.2 after the project period. A process definition that included transition procedures could have led to the next process, including a review of the draft guidance. However, the University of Tokyo project may not have progressed owing to a lack of evidence, as the opinion paper states, “further consideration is required for biomarker selection.”

In the GoG type DDTQP, by defining the process from Steps 2 and 3 of the Analytical Framework, the activities in the process are controlled, and tool guidance is drafted efficiently. Contrastingly, IFDIP projects were managed under a non-GoG type system, and there were no clear criteria for the completion of each step and/or procedures for the next step.

During the step-by-step transition from Transition 1 to 3, the project leader must access the MHLW section in charge, including the review team candidates, and discuss the adequacy of the data. However, regulators inside the team are often not members of the formal review team, and the transition procedures are not explicitly defined. Therefore, the progress of the project depends on the PL’s personal competence.

Furthermore, non-GoG systems, such as the IFDIP, do not guarantee the formation of a review team. Although the necessary data packages have been submitted to the grant office, the review process has not yet been disclosed. The progress of each step in the DDTQP is disclosed. However, in the case of the IFDIP, there is a disclosure of annual reports, but none from the regulator after the grant period ends. Thus, there is no method for progress management after Transition 1 on the tool owner’s side.

In these cases, for an excellent project team with a regulatory expert network, tool guidance is issued even under a non-GoG type system. However, for a normal project team, delays and stagnation often occur under non-GoG type systems. This suggests the advantage of the GoG type tool qualification system.

### Requirements for Guidance Configuration and Quality

The contents of deliverables generated through the IFDIP vary greatly, depending on whether they are for a single tool or set COUs for a single product or disease. However, the guidance in the DDTQP is for the combination of a single tool and a single COU, making the configuration simpler.

Another difference is that the IFDIP does not directly post evidence in the tool guidance document but instead cites references, whereas DDTQP always attaches evidence data to guidance. Thus, the guidance items were standardized during the process. Mandating that the evidence data shown in the guidance facilitate access to the underlying evidence is a requirement for tool guidance.

Although the IFDIP allows for a variety of forms of output, to avoid a situation in which tool guidance is not issued despite sufficient evidence, there is a need to identify the recommended tool guidance components, including determining the type and level of evidence sufficient to support qualification.

For example, the Evidentially Framework for Biomarkers in DDTQP [53] and the APPRAISAL OF GUIDELINES FOR RESEARCH & EVALUATION II (AGREE II) [54], which provide guidance for evaluating the quality of clinical guidance, could be good references for framing the components of tool guidance.

### Connection from R&D to Qualification

Regulators were incorporated into the project team in the non-GoG version of the IFDIP. The advantage of the IFDIP is that the regulator can be involved in the research plan from Step 1 and provide continuous consultation until the acquisition of data for the validation items required in Step 2. Contrastingly, in the DDTQP, the FDA organizes a review team and is involved in the research plan from Step 2 onwards. The IFDIP-type grants can be combined with DDTQP to take advantage of both. A Funding Opportunity Announcement (FOA), a grant to support research to continue the development of drug development tools with an approved LOI within the DDTQP, was initiated by the FDA in FY2021 [55]. The combination of tool qualifications and grants is also expected to increase in the near future.

## Conclusion

To compare the GoG and non-GoG type tool qualification systems, we constructed an analysis framework that generally defined the process of tool qualification and its pre- and post-processes and analyzed Japanese non-GoG type cases with the control of a US GoG type case.

Although tool guidance could have been issued under the non-GoG system with a regulatory expert network on the team, we confirmed cases in which tool qualification could have been insufficient because the process transition procedure was not clear without a process definition and the conditions for transition, suggesting that the GoG type system may promote tool qualification more effectively.

From the content analysis of the guidance, while the DDTQP has a one-to-one relationship between the tool and the COU, the IFDIP’s tool guidance, which allows for diverse relationships between tools and target indications, was inconsistent in structure. This observation led to the need to develop horizontal requirements to maintain a certain level of composition and quality in tool guidance, covering both single and multiple tools.

## Data Availability

All data are in the articles listed in the reference list.
